# Mucosal vaccines and technology

**DOI:** 10.1111/cei.13285

**Published:** 2019-04-08

**Authors:** A. Miquel‐Clopés, E. G. Bentley, J. P. Stewart, S. R. Carding

**Affiliations:** ^1^ Gut Microbes and Health Research Programme Quadram Institute Bioscience Norwich UK; ^2^ Department of Infection Biology University of Liverpool Liverpool UK; ^3^ Norwich Medical School University of East Anglia Norwich UK

**Keywords:** bacterial, mucosal, vaccines, viral

## Abstract

There is an urgent and unmet need to develop effective vaccines to reduce the global burden of infectious disease in both animals and humans, and in particular for the majority of pathogens that infect via mucosal sites. Here we summarise the impediments to developing mucosal vaccines and review the new and emerging technologies aimed at overcoming the lack of effective vaccine delivery systems that is the major obstacle to developing new mucosal vaccines.

## Introduction

Vaccination is an efficient and cost‐effective form of infectious disease prevention that can lead to global eradication, as seen for smallpox (1980) and rinderpest (2011). However, there is an urgent and growing need for the development of new and improved vaccines to further reduce the global burden of infectious disease morbidity and mortality, particularly against those targeting the respiratory and gastrointestinal (GI) tract. The paucity of effective vaccines is also acute in veterinary medicine, which is compounded by increasing multi‐drug and antibiotic resistance 1. Vaccines to combat zoonoses are a particularly urgent priority, as 60% or more pathogens with the potential to harm humans originate in animals 2. Current vaccines are delivered by injection with associated problems of safety, compliance, morbidity and the high cost of mass immunization, particularly in resource‐poor developing countries. Injected vaccines also provide partial or no protection at mucosal sites. Considering that >90% of pathogens gain access to the body via mucosal sites, using mucosal vaccination to generate protective immunity at mucosal sites could overcome the limitations of current injection‐based vaccines in providing front‐line protection against pathogen invasion and dissemination 3. However, only a handful of mucosal vaccines are currently licensed. This limited availability of mucosal vaccines is related to the lack of effective delivery systems able to preserve vaccine antigen integrity and strong adjuvanticity, which is compounded by the intrinsic nature of the mucosal immune system to induce tolerance 4.

## Mucosal immunity and vaccine responses

The majority of mucosal vaccines are administered by the oral and nasal routes with the vaginal, rectal, ocular and sublingual routes being less frequently used. However, not all routes of administration induce an equivalent immune response in terms of potency and longevity, reflecting differences in the organization and cellular make‐up of lymphoid structures in different mucosal tissues 5, 6. For example, oral immunization usually stimulates immune responses in the GI tract in addition to the oral mucosa and nasal‐associated lymphoid tissues (NALT) and mammary glands. Intranasal delivery effectively induces antibody production in salivary glands, the NALT and the bronchus‐associated lymphoid tissue (BALT) of the lower respiratory tract, and in the urogenital tract. Rectal immunization elicits a more pronounced immune and antibody response in nasal secretions, tears and the rectal mucosa. Thus, depending on the mucosal sites targeted by different pathogens, the route of immunization needs to be carefully considered 6, 7, 8. In most cases, mucosal vaccination is also effective in priming systemic immune responses and generating serum antibodies with neutralizing properties, reflecting the cross‐talk between the mucosal and systemic immune systems. Serum immunoglobulin (IgG) responses in vaccinated animals can be a useful correlate of protection, either alone or in combination with secretory mucosa‐derived (IgA) antibodies 9.

Size is another important consideration in the design of mucosal vaccines and targeting uptake to inductive immune sites to generate T cell and/or B cell responses. Goblet cell‐associated passageways allow the entry of soluble protein antigens, but not inert particles (0·02–2 µm) into the underlying lamina propria 10. Via endocytosis, enterocytes readily take up nanosized soluble particles of 20–40 nm, whereas M cells are the major route by which larger‐sized inert particles of more than 100 nm are taken up 11. While nanoparticles lead predominantly to T cell responses, larger microparticles are more effective at inducing humoral responses 12, 13. The fate of particles delivered via the intranasal route is influenced by their size with particles smaller than 5 µm being transported across the nasal mucosa for delivery to cells of the BALT. By contrast, larger particles exceeding 10 µm are taken up by alveolar macrophages and dendritic cells (DCs) 14, 15.

## Current licensed mucosal vaccine formulations

### Human use

The majority of currently licensed human mucosal vaccines comprise attenuated strains of pathogenic bacteria or viruses that retain their immunogenicity during transit through the upper GI tract and can target inductive immune sites in the small and large intestine or upper respiratory tract (Table 1). The oral polio vaccine, OPV, is the most successful mucosal vaccine to date 16. A significant drawback to using attenuated pathogen‐based vaccines is the risk of reactogenicity and reversion to a virulent pathogen following vaccination, usually in immunocompromised infants, elderly people or in individuals with a specific immunodeficiency. Although the use of OPV has decreased the number of polio cases by more than 99% since 1988, there are still disease outbreaks of vaccine‐associated paralytic polio (VAPP) that arise due to small genetic changes occurring during OPV replication in humans 17, 18. Another concern for live attenuated vaccines is the possibility of retrograde transport to the brain after nasal or intranasal vaccination, as happened with a replication‐defective adenovirus vector carrying three proteins from human immunodeficiency virus type 1 that was found in the central nervous system of mice after intranasal delivery, which may have reached the brain via olfactory neurones 19.

**Table 1:** Licensed mucosal vaccines

Target	Pathogen	Trade name	Delivery route (form)	Formulation (±adjuvant)
Human	*Vibrio cholerae*	Dukoral^®^	Oral (liquid)	Inactivated (recombinant cholera toxin subunit B)
		ShanChol^®^, Euvichol^®^	Oral (liquid)	Inactivated
		Vaxchora^®^	Oral (liquid)	Live attenuated
	Influenza type A and B virus	FluMist™	Intranasal (spray)	Live attenuated
	Poliovirus	Biopolio™ B1/3, and other oral polio vaccines – OPVs	Oral (liquid)	Live attenuated
	Rotavirus	Rotarix^®^ and RotaTeq^®^	Oral (liquid)	Live attenuated
	*Salmonella typhimurium*	Typhi Vivotif^®^	Oral (capsules)	Live attenuated
	Adenovirus	Not trade name	Oral (tablets)	Live attenuated
		Approved for military		
Animal	Rabies virus	RABORAL‐V‐RG	Oral (bait)	Recombinant (Vaccinia virus vector)
	Bovine parainfluenza 3	Rispoval	Intranasal (spray)	Live attenuated
	bovine respiratory syncytial virus			
	*Bordetella bronchiseptica*	Nobivac^®^	Intranasal (drops)	Live
	Canine parainfluenza virus			
	Newcastle disease virus	Avinew NeO™	Oral, ocular or nasal (spray, drinking water or drops)	Live attenuated

Apart from live attenuated vaccines, three World Health Organization (WHO) prequalified inactivated oral vaccines are in use for cholera (Dukoral^®^, Shanchol™ and Euvichol^®^), which have been shown to provide high levels (60–95%) of long‐lived (>2 years) protection in support of the concept that non‐living vaccines can be effective for mucosal delivery and vaccination 20. Although inactivated vaccines are, in general, safe, the process of inactivation (heat and/or formalin treatment) can reduce their immunogenicity and require the addition of adjuvants such as recombinant cholera toxin subunit B, which is included in the Dukoral vaccine 20.

Subunit vaccines comprising synthetic recombinant peptides and proteins, toxoids, DNA or mRNA offer significant safety advantages over attenuated and inactivated vaccines. They are inert and non‐infectious, although they can suffer from weak immunogenicity and a requirement for adjuvants. To date, subunit vaccine formulations have failed to confer long‐lived protective mucosal immunity in humans 21. The weak immunogenicity of inert molecules and protein subunit antigens after delivery to mucosal sites is due in large part to their inefficient uptake by the mucosal epithelium and delivery to the delivery to the mucosa‐associated lymphoid tissue (MALT) 22, 23. This reflects the significant physical, biochemical and microbial obstacles orally and nasally administered vaccines must overcome in the GI and respiratory tracts in order to access and activate mucosal immune cells. During transit through the GI tract, vaccine antigens are diluted and can be retained or trapped in mucosal secretions and by mucus and be subsequently degraded by non‐specific host or microbial enzymes prior to reaching the mucosal immune system. The acidic environment of the upper GI‐tract also impacts on the stability and integrity of oral vaccines 24. In the respiratory tract, physical discharge due to high mucocillary clearance rates, or peristalsis action in the GI tract, also impact upon vaccine integrity and retention time 25.

### Veterinary use

Mucosal vaccines have been more successful in the veterinary field, with spray and drinking water vaccines routinely used for mass vaccination in poultry farming. Recent introduction of edible gel‐bead‐based vaccine systems offer a more stable mucosal delivery, protecting live vaccines against environmental inactivation to improve bioavailability. Use of gel‐beads to deliver *Eimeria* spp. oocysts to day‐old chicks offers greater uptake of oocysts than water spray containing *Eimeria *spp. oocysts, and significantly higher weight gain post‐challenge infection 26. The livestock–wildlife interface consistently poses difficulties in vaccination programmes for animals. Mucosal delivery of vaccines through baiting allows free‐ranging animals to voluntarily uptake vaccines, in order to break down interspecies transmission of infectious disease between wild and domesticated animals. Arguably, the most successful bait vaccine is RABORAL V‐RG^®^ which, following distribution into wildlife habitats, has aided eradication of wildlife rabies from Belgium, France and Luxembourg 27. Wildlife bait vaccination has also helped to control other pathogens, including classical swine fever in wild boar (*Sus scrofa*) in Europe 28 and plague in prairie dogs (*Cynomys *spp.) in the United States 29, 30. Currently, licensed vaccines for parenteral application could be administered orally where it may not be possible, or feasible, to trap an animal to inject them. *Mycobacterium bovis* is a causative agent of tuberculosis (TB), and remains one the most difficult diseases of livestock to control, due largely to the prevalence of a wildlife reservoir. Bacillus Calmette–Guérin (BCG) vaccines were developed to protect cattle against bovine tuberculosis with subsequent experimental and field studies, showing that they may be efficacious in the control of *M. bovis* in wild animals after mucosal administration. White‐tailed deer (*Odocoileus virginianus*) vaccinated with BCG Danish strain 1331 by oral bait or oral liquid had fewer tuberculosis lesions 5 months post‐*M. bovis* challenge than control deer 31. Badger BCG is a licensed injectable vaccine for European badgers (*Meles meles*) against TB; however, capturing animals for injection is labour‐intensive and stressful. Oral administration of BCG has been shown to reduce *M. bovis* lesions in badgers 32, with 75% of captured badgers in a further study testing positive for BCG vaccine markers where the vaccine was administered in bait 33. Dispersing mucosal vaccines in baits into high‐risk areas could help to reduce endemic TB in wildlife reservoirs, reducing the risks of devastating TB outbreaks in livestock. Despite promise from field trials, there is still a lack of vaccines licensed for distribution into wildlife. A major drawback is the risk of non‐target species consuming the bait; however, with further research into the use of subunit or inactivated mucosal vaccines, instead of live, this threat may be withdrawn.

## The need for human mucosal vaccines

Despite many trials, there are no licensed vaccines for many human mucosal‐transmitted pathogens (Table 2), or the currently available vaccines generate incomplete protection.

**Table 2:** Infectious diseases in need of mucosal vaccines

Pathogen	Mortality/annum	Morbidity/annum	Ref.
**Respiratory tract**			
Seasonal influenza	470 000	4 million	34
RSV‐ALRI	128 000	33·8 million	35
*Streptococcus pneumoniae*	1·6 million		36
*Mycobacterium tuberculosis*	1·6 million	10 million	37
**Gastrointestinal tract**			
Rotavirus	215 000		38
*Helicobacter pylori*	14 500		39
Enterotoxigenic *Escherichia coli* (ETEC)	400 000		40, 41
*Salmonella*	32 000 (Africa)	1 2 million (USA)	42, 43
*Shigella*	700 000	80 million	44
*Clostridium* (*difficile*/*perfringens*)	14 000	500 000	45
**Urogenital tract**			
Syphilis	205 000		46
Gonorrhoea		78 million	47
Herpes simplex virus 2		417 million	48
Human papillomavirus (HPV)	270 000		49
Hepatitis B	887 000		50
Hepatitis C	399 000	71 million	51
HIV	940 000	36·9 million	52

## Improving mucosal vaccines

New technologies are being developed with the aim of protecting and preserving antigen structural integrity, as well as increasing bioavailability and induction of local and systemic neutralizing immune responses (Table 3). All these delivery vehicles can be modified or complemented with immunostimulatory molecules or coating agents [e.g. polyethylene glycol (PEG), chitosan] to change their charge, adhesive properties, shape, size and/or pH to improve their characteristics, interactions with host cells and targeting sites of inductive immune responses. The incorporation of PEG into polyactide (PLA) nanoparticle vaccine formulations has been shown to be effective for the oral delivery of hepatitis B surface antigens in mice 53. Chitosan is a biodegradable, biocompatible, muco‐adhesive, non‐toxic polymer that has been used in a similar way to protect *Escherichia coli *O157:H7 vaccine formulations during oral delivery 54.

**Table 3:** Novel mucosal vaccine delivery systems

Delivery system	Structure	Advantages	Limitations	Ref.
Liposomes	Spherical phospholipid bilayer entrapping an aqueous solution core	Ease of incorporating distinct types of antigens Adaptable physicochemical properties Lepidic compounds with adjuvant properties	Relatively low intrinsic stability for storage and after administration Potent toxicity of cationic lipids (dose‐dependent)	55
Archaeosomes	Liposomes composed of Archaea‐derived polar lipids	Stable formulations Improved immunogenicity compared with liposomes	Preparation and purification of Archaea lipids Need optimization of production and formulation	56
Bilosomes	Bile salt stabilized vesicles	Stable in gastric environment High stability	Relatively low antigen dose	57
ISCOM, ISCOMATRIX	Cage‐like structure comprised of cholesterol, phospholipids and saponin	Composition, size and surface structure like virus Self‐adjuvanticity due to saponin	Inclusion of antigens into the ISCOM can be difficult	58
Bacterial outer membrane vesicles (OMV)	OMVs from Gram‐negative pathogens containing microbe‐associated molecular pattern (MAMPs) and membrane proteins	In‐built adjuvanticity High stability over a wide range of temperatures and pH Safe use in children and adults and effective in controlling disease outbreaks	Chemical detoxification required – reduced adjuvanticity Variable efficacy Strain‐specific – limited heterotypical strain protection	59, 60
Virus‐like particles (VLP)	Natural virus without carrying genetic material	Highly immunogenic without addition of adjuvant Antigens can be chemically conjugated or genetically inserted	Purification can be a challenge May have poor quality and consistency Contamination by host materials	61, 62
Gene gun (DNA vaccination)	DNA‐coated colloidal gold particles	Fast and simple Efficient DNA transduction Requires small amounts of DNA (0·1 mg/dose) Can be used to deliver multiple DNAs	Costly device and reagents Limited to exposed tissues Depth of penetration *versus* tissue damage Preferentially induces T helper type 2 response Multiple factors influence efficacy	63
Emulsions Water‐in‐oil/ oil‐in‐water	Nanosized droplets	Slow release of immunogen Ease of manufacture Self‐adjuvanticity	Reactogenicity Limited stability after administration Low preservation of antigen structure	64, 65
Synthetic polymer nanoparticles (e.g. PLA/PLGA)	Polylactide (PLA) or polylactic‐co‐glycolic acid (PLGA) based nano‐ and micro particles FDA‐approved agents	Controlled release of antigens Biodegradable and biocompatible biopolymer	Sensitivity to harsh gastric environment, low loading capacity	66, 67
Natural polymer nanoparticles (e.g. chitosan)	Chitosan based nano‐ and microparticles	Biocompatible, biodegradable, mucoadhesive and immunostimulating	Irregular distribution, low physical stability	68
Hydrogel (e.g. cCHP nanogel)	Cationic cholesterol‐bearing pullulan nanogel, self‐assembles with water due to their amphiphilic polysaccharides	Ability to function as an artificial chaperone Prolonged binding to nasal epithelium	Optimization of biodistribution and degradation mechanism Component toxicity	69
Lactic acid bacteria (LAB)	Live recombinant LAB expressing antigens Generally recognized as safe (GRAS)	Easy and safe production and storage Survives gastric environment Self‐adjuvanticity	Safety concerns using genetically modified organisms	70
Chemically processed pollen grains (PGs)	Resistant bilayer pollen grain shell	Self‐adjuvanted Protected from harsh environment	Chemical treatment methods required to eliminate allergens from pollen grain	71, 72
Terrestrial plants and algae	Plant or algae cells with an antigen created by gene modification	Highly resilient cell wall Easy manufacturing process and scale‐up Suitable for mass vaccination No cold chain requirement	Use of transgenic plants and regulatory body approvals	73, 74, 75

Modified from Corthesy *et al*. 54.

The inclusion of alginate, polyvinyl alcohol, hyaluronan and cellulose to micro‐ and nanoparticle‐based vaccines increases their viscosity and augments the retention time at mucosal surfaces promoting antigen uptake. Molecules that target the carrier or vaccine antigen directly to surface receptors on M cells or antigen‐presenting cells [e.g. Toll‐like receptors (TLRs)] have also been used 76. The use of adjuvants includes aluminium hydroxide to facilitate antibody and T helper type 2 (Th2) CD4 T cell responses, or *Vibrio cholerae* toxin (CT) and heat‐labile enterotoxin from *E. coli* to non‐specifically boost cellular and humoral immune responses 77, 78. To date, recombinant cholera toxin subunit B (rCTB) is the only adjuvant accepted for inclusion in licensed mucosal vaccines (i.e. Dukoral^®^ vaccine). rCTB stimulates the production of both anti‐bacterial and anti‐toxin antibodies without any side effects 79. Genetically modified enterotoxins are being developed to reduce toxicity without adversely affecting their adjuvanticity.

Plants can be used as bioreactors to produce large quantities of vaccine that are then purified from plant extracts or can be consumed directly as an edible plant vaccine. The plants of choice are rice, lettuce or maize, with an edible rice‐based cholera vaccine containing rCTB (MucoRice‐CTB) currently in Phase I clinical trials 80. An experimental lettuce‐based hepatitis B virus vaccine has been tested in mice and shown to be effective at inducing neutralizing antibodies after oral administration 81. Algae are a particularly cost‐effective bioreactor option for producing large quantities of recombinant vaccines, and due to their high structural integrity and resilience of their cell walls have the potential to be used intact as vaccine delivery vehicles 74. *Chlamydomonas reinhardtii* has been used in experimental *Staphylococcus aureus* vaccine formulations 82 and *Schizochytrium *sp. have been used to develop novel zika virus vaccines 83; in both cases, these algae‐based vaccines have been shown to be effective at eliciting both mucosal and systemic humoral immune responses.

Immunostimulatory complex (ISCOM) technology has been incorporated into commercial veterinary vaccines such as Equip F^®^ vaccine against equine influenza for parenteral delivery, although Ghazi *et al*. have demonstrated protection in mice immunized orally with influenza virus subunit vaccines that incorporate ISCOM 84. Liposomes and emulsions carriers have been used in experimental vaccines for respiratory virus infections with incorporation of the soybean oil‐based emulsion W805EC into influenza virus and inactivated respiratory syncytial virus (RSV) vaccines, both of which are effective at eliciting protective systemic and mucosal antibody immune responses, and RSV vaccine also induces cellular immunity after intranasal administration 85, 86. Similarly, pneumococcal surface protein A (PspA)‐based subunit vaccines incorporating a cCHP‐based nanogel has been shown to induce both mucosal and systemic neutralizing antibodies in cynomolgus macaques after nasal delivery 87.

Virus‐like particles (VLP) are an attractive option as a vaccine delivery vehicle due to their useful properties, such as the ability to induce adaptive immune response and to induce long‐term expression of non‐self‐proteins 88, 89. VLPs from adeno‐associated viruses (AAV) have been used to develop novel influenza virus vaccines encoding camelid‐derived anti‐influenza antibodies transgenes that when administered intranasally protected mice against lethal influenza A and B challenge 89, 90. Other preclinical studies using AAV as a carrier include norovirus vaccine formulations containing viral protein and RNA, that have shown promise in a Phase I clinical trial 91, and a chimpanzee‐derived AAV expressing hepatitis C virus antigens that is currently in Phase II clinical trials 92, 93.

Gene gun bombardment is a biolistic system for mucosal DNA vaccination. This needle‐free technology is based on propelling DNA‐coated colloidal gold microprojectiles at exposed tissue surfaces (e.g. skin, vulva and mouth) and penetrating the cytosol and cell nucleus of cells within deeper layers of the tissue. 94 Epidermal DNA vaccines delivered via a gene gun have been shown to elicit both humoral and cell‐mediated mucosal immune responses in experimental animals and cattle 63, 95, 96. To improve the potency of immune responses gene to gun gene immunizations, DNA can be combined with adjuvants such as recombinant protein antigens and plasmids encoding cytokines 94, 97. However, gene gun‐mediated delivery has limited or no control over where and how effective DNA transduction is in host cells and is generally ineffective at inducing immune responses of sufficient potency to provide effective and long‐lived protection.

The use of genetically modified probiotic strains of bacteria to deliver vaccine antigens has been explored for human papilloma virus (HPV) vaccines. Although a commercial HPV vaccine is available, it does not confer protection to all HPV‐related cancers 49. Generally recognized as safe (GRAS) strains of *Lactoccus lacti* engineered to express the HPV‐16 E6 oncoprotein generated humoral and cellular immune response in mice after oral administration, as well being shown to have an inhibitory effect on tumour growth 98. There are, however, biosafety and environmental contamination concerns in using genetically modified bacteria 99.

A safer alternative to using viable bacteria as vaccine delivery vehicles are non‐viable nanometer‐sized lipid‐containing microvesicles (outer membrane vesicles; OMVs) that are naturally produced and secreted by most Gram‐negative bacteria 59. Formulations of *Neisseria meningitidis* OMVs (VA‐MENGOC‐BC, MenBvac, MeNZB and Bexero) have been successfully used to vaccinate both adults and children and to control outbreaks of meningococcal B infection in several countries 100, 101. OMVs from other Gram‐negative pathogens are also promising vaccine candidates, including those from *Salmonella* 102, *Shigella flexneri* 103 and *V. cholerae* 104. However, their potential for unintended toxicity due to associated toxins is a safety concern and limits their widespread use, although chemical detoxification can overcome this, but at the loss of immunogenicity and adjuvanticity 105. In principle, these limitations could be overcome by bioengineering the parental bacterium to improve their OMV drug‐delivery capability 60. Alternatively, non‐pathogenic commensal bacteria could be used as a source of OMVs to reduce toxicity and improve safety. We are developing an OMV‐based drug and biologicals delivery technology platform based on the use of OMVs produced by strains of human commensal *Bacteroides* engineered to express in their OMVs bacterial or viral vaccine antigens or human therapeutic proteins for delivery to the respiratory and GI tracts.

In summary, while mucosal vaccines represent the ideal means of protecting against the majority of infections, there are very few licensed vaccines for either humans or animals. A raft of new technologies and innovations in vaccine antigen encapsulation and delivery are being developed to overcome the obstacles of protecting and preserving antigen structural integrity as well as increasing bioavailability and induction of local and systemic neutralizing immune responses during transit to mucosal inductive immune sites, particularly in the GI tract.

## Disclosures

The authors report no conflicts of interest.
